# The role of FKBP5 in cancer aetiology and chemoresistance

**DOI:** 10.1038/sj.bjc.6606014

**Published:** 2010-11-30

**Authors:** L Li, Z Lou, L Wang

**Affiliations:** 1 Department of Molecular Pharmacology and Experimental Therapeutics, Mayo Clinic, Rochester, MN 55905, USA; 2 Division of Oncology Research, Department of Oncology, Mayo Clinic, Rochester, MN 55905, USA

**Keywords:** FKBP5, immunophilins, chemoresistance

## Abstract

FK506 binding protein 51 (FKBP51, also called FKBP5) belongs to a family of immunophilins, FK506 binding proteins (FKBPs). Members of this family are targets for drugs such as rapamycin and cyclosporine. Although FKBP5 shares characteristics with other FKBPs, it also has unique features, especially its role in the regulation of multiple signalling pathways and in tumourigenesis and chemoresistance. In this review, we will focus on the recently discovered role of FKBP5 in cancer aetiology and response to antineoplastic therapy.

FK506 binding protein 51 (FKBP51, also called FKBP5) is a 51-kDa FK506 binding protein that is a member of a family of immunophilins, FK506 binding proteins (FKBPs). Members of this family contain both FKBP domain(s) and tetratricopeptide repeat (TPR) domain(s) (Kang *et al*, 2008). The FKBP domain contains peptidylprolyl isomerase (PPIase) activity that catalyses the *cis*–*trans* conversion of peptidylprolyl bonds, a reaction that is important for protein folding (Fruman *et al*, 1994). The TPR domains at the C terminus are involved in protein–protein interactions (Scheufler *et al*, 2000). In general, TPR domains are all-helical structures consisting of 2–16 units of a consensus 34-aa motif. The domain is found in more than 800 different proteins from bacteria and humans. The first described and most well-known FKBP family member is FKBP12, a small protein with a single FKBP domain and a molecular size of 12 kDa. FK506 binding protein 12 is a major binding partner for FK506 and rapamycin (Siekierka *et al*, 1989). Additional members of this family have been identified on the basis of their ability to bind FK506, including FKBP13, FKBP25, FKBP38, FKBP52, and FKBP51 (named to reflect their molecular weights), all of which contain the FKBP domain that is responsible for drug ligand binding and PPIase activity (Kang *et al*, 2008).

Human FKBP5 was first cloned from a HeLa cell cDNA library in 1995 (Baughman *et al*, 1995). It mediates FK506 inhibition of calcineurin *in vitro* and is highly expressed in murine T lymphocytes (Baughman *et al*, 1997). FK506 binding protein 5 contains two consecutive FKBP domains and a three-unit repeat of the TPR domain (Figure 1). The first FKBP domain (FK1) of FKBP5 shares 48% sequence identity to the FK domain of FKBP12 and has measurable PPIase activity, with a binding pocket for FK506 and rapamycin (Sinars *et al*, 2003). FK2 is also structurally similar to the FKBP domain of FKBP12, despite having only 26% sequence identity. However, FK2 lacks measurable PPIase activity. Presumably, FK2 resulted from an FK domain duplication event, but subsequently lost its PPIase activity, while it appeared to have gained protein interaction ability (Sinars *et al*, 2003). The FKBP5 structure study provides important initial insights into the relative orientations of the FK1, FK2, and TPR domains that are important for protein interaction and/or drug ligand binding (Sinars *et al*, 2003).

One of the major functions of FKBP5 is its involvement in the modulation of steroid receptor function, including progesterone, androgen and glucocorticoid receptors (GR), by forming a complex with the heat shock proteins Hsp90/Hsp70. Both FKBP5 and its family member FKBP4 (also called FKBP52) are involved in steroid receptor signalling, and their interchange served as the earliest known event in steroid receptor activation. Davies *et al* (2002) have used both *in vitro* and *in vivo* models to show the hormone-induced loss of FKBP5 and gain of FKBP4, providing evidence that the hormone-binding event causes an interchange of FKBP5 and FKBP4 immunophilins within the GR heterocomplex. The complex then dissociates, allowing the binding of GR to DNA-binding motifs in its target genes to regulate gene transcription (Figure 2A). Therefore, FKBP5 has been identified as a sensitive biomarker of corticosteroid responsiveness *in vivo* (Table 1) (Sinars *et al*, 2003; Rees-Unwin *et al*, 2007). In addition to a role in tumourigenesis (see below), numerous studies have also indicated that FKBP5 may have a role in psychiatric diseases such as depression and in response to the treatment of depression through modulation of hormone receptors (Schosser and Kasper, 2009). FK506 binding protein 5 and Hsp90 are also important for the clearance of tau, a microtubule-associated protein that accumulates in a group of neurodegenerative disorders such as Alzheimer's disease (Jinwal *et al*, 2010).

Of equal or greater interest than these effects on steroid hormone signalling are recent studies that have indicated that FKBP5 could also be a biomarker for tumourigenesis and chemoresistance. Roles in those processes extend beyond its functions as a co-chaperone and a PPIase. Subsequent paragraphs will focus on the discovery of the role of FKBP5 in cancer aetiology and chemoresistance.

## FK506 binding protein 51 expression and regulation in cancer

Human FKBP5 is highly expressed in multiple tissues, including kidney, skeletal muscle, liver, placenta, heart, and peripheral blood. However, its expression level is much lower in the pancreas, spleen, and stomach (Baughman *et al*, 1997). Although initial studies found no detectable FKBP5 in the brain, colon, or lung, subsequent studies have shown expression of FKBP5 in human brain tissues (Nair *et al*, 1997) and colon tissues (Mukaide *et al*, 2008). FK506 binding protein 5 expression can be induced through the activation of progesterone receptor, androgen receptor (AR), and GR (Ratajczak *et al*, 2003). Hormone response elements in the introns of FKBP5 regulate the induction of expression by glucocorticoids, progesterone, and androgens. For example, Febbo *et al* (2005) had identified FKBP5 as an androgen-inducible gene, and demonstrated a physical interaction between FKBP5 and AR. Recent studies have identified a long-range activation of FKBP5 transcription in prostate cancer cells by the AR via distal intronic enhancers (Makkonen *et al*, 2009). The FKBP5 locus harbours 13 *in silico*-predicted androgen response elements (AREs), most of which are located downstream from the transcription start site and are capable of binding AR *in vitro*. These results indicated that very distal AREs can act as robust enhancers, highlighting the potential importance of the long-range regulation of FKBP5 expression by the AR (Makkonen *et al*, 2009). FK506 binding protein 5 expression can also be induced by the GR in human lung cancer A549 cells, where FKBP5 mRNA are accumulated in response to dexamethasone exposure (Paakinaho *et al*, 2010). A major intronic enhancer within the *FKBP5* locus was identified to bind to GR. There were also GREs identified in the proximal promoter of *FKBP5*. The GR is capable of activating FKBP5 transcription and also evoking changes in chromatin structure (Paakinaho *et al*, 2010). FK506 binding protein 5 can also function within an auto-regulatory negative feedback loop to modulate its own expression. Exogenous FKBP5 expression was able to downregulate the transcription of genomic FKBP5 in the presence of dexamethasone through this auto-regulation process (Park *et al*, 2007).

Both overexpression and downregulation of FKBP5 have been observed in human cancers. According to the Oncomine, FKBP5 is overexpressed in brain cancers, prostate cancer, lymphoma, head and neck cancer, and melanoma. On the other hand, FKBP5 has also been shown to be downregulated in pancreatic cancer (Pei *et al*, 2009), melanoma, colon cancer, and testicular cancer. Gene expression data for those studies are available through the Oncomine, and studies describing these observations are also referenced in this website (http://www.oncomine.org). Similar to its expression pattern, FKBP5 has been shown to either promote or suppress tumour growth through its regulation of different signalling pathways in a specific tissue environment.

## Role of FKBP5 in tumourigenesis and response to antineoplastic therapy

**Figure 2:**
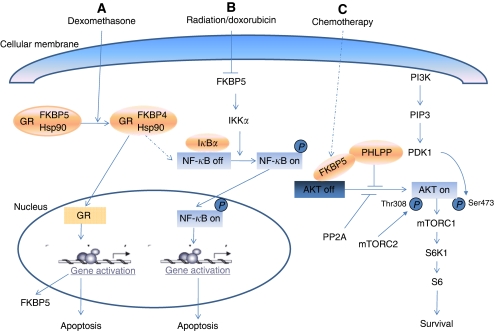
Schematic model of FKBP5 functions involved in several different signalling pathways, including glucocorticoid receptor (GR) signalling pathways (**A**), as well as NF-*κ*B (**B**) and AKT–PHLPP (**C**) pathways. (**A**) In the absence of dexamethasone, FKBP5 is the primary immunophilin of the FKBP–Hsp90–GR complex in an inactive stable state. Upon dexamethasone binding, FKBP5 is displaced by FKBP4, and the complex can enter the nucleus. The complex then dissociates, allowing binding of GR to DNA-binding motifs of target genes, leading to apoptosis. (**B**) In the presence of doxorubicin or damage from radiation, FKBP5 might involve in the control of IKK*α* activity, which can induce I*κ*B*α* degradation via its phosphorylation, nuclear translocation, and activation of NF-*κ*B, and the expression of its target genes, consequently triggering cell apoptosis. (**C**) FK506 binding protein 5 interacts with PHLPP and AKT, acting as a scaffolding protein that promotes the interaction between AKT and PHLPP, thereby enhancing the dephosphorylation of AKT and inactivating AKT, which results in the blockade of AKT signalling for cell survival and leads to cell apoptosis or death.

FK506 binding protein 5 has been shown to be involved in several different signalling pathways, including steroid receptor signalling pathways, NF-*κ*B, and AKT (a serine/threonine protein kinase, also called PKB)–PHLPP (leucine-rich repeat protein phosphatase) pathways, all of which have important roles in tumourigenesis and response to antineoplastic chemotherapy (Figure 2 and Table 1).

### FK506 binding protein 5 is a key regulatory component of the androgen–receptor complex

Although FKBP5 knockout mice show normal androgen signalling (Yong *et al*, 2007), it has been linked to AR signalling and prostate cancer. As discussed above, FKBP5 itself is a target gene of AR. FK506 binding protein 5 can form a complex with AR to enhance AR transcriptional activity in prostate cancer (Febbo *et al*, 2005). Recently, Ni *et al* (2010) showed that FKBP5 stimulates the recruitment of cochaperone p23 to the ATP-bound form of Hsp90, forming an FKBP5–Hsp90–p23 superchaperone complex, which stimulates androgen-dependent transcription activation and cell growth. Therefore, FKBP5 might serve as a novel drug target to help disrupt AR-mediated signalling in prostate cancer.

### FK506 binding protein 5 regulates the NF-*κ*B pathway

Bouwmeester *et al* (2004) first identified a physical interaction between FKBP5 and nuclear factor I*κ*B kinase *α* subunit (IKK*α*) during a proteomics study. In that study, FKBP5 was copurified with IKK*α* as well as IKK*ε*, TGF beta activated kinase 1, and MAP kinase/ERK kinase kinase 1 (Bouwmeester *et al*, 2004). The interaction with IKK*α* was confirmed by co-immunoprecipitation. These results indicated that FKBP5 might have a role in NF-*κ*B signalling.

Several studies by Romano *et al* have demonstrated that the downregulation of FKBP5 can sensitise cells to irradiation and anthracyclines through the regulation of NF-*κ*B (Avellino *et al*, 2005). They showed that treatment of melanoma or acute lymphoblastic leukaemia cell lines with anthracycline would induce NF-*κ*B activity. However, treatment with rapamycin significantly sensitised the cells to anthracycline by inhibition of the NF-*κ*B activity. This effect was associated with FKBP5 activity, as downregulation of FKBP5 increased the rapamycin inhibition of NF-*κ*B activity, and, in turn, increased apoptosis (Avellino *et al*, 2005). In addition, the same group also found that higher expression of FKBP5 might influence radioresistance through the activation of NF-*κ*B in melanoma cells (Romano *et al*, 2010). They also found that FKBP5 was overexpressed in melanoma samples compared with normal skin tissue, and pretreatment of mice xenograft tumours with FKBP5-siRNA provoked massive apoptosis after irradiation (Romano *et al*, 2010). These experiments provided evidence that, both *in vitro* and *in vivo*, FKBP5 might be a promising target for radiosensitising agents against malignant melanoma.

### FK506 binding protein 5 is a negative regulator of AKT activation in tumourigenesis and chemoresistance

Regulation of NF-*κ*B by FKBP5 emphasised the potential importance of FKBP5 in tumourigenesis and response to therapy. However, it was not until very recently that another important function of FKBP5 was identified. Studies from our laboratory first demonstrated, through the use of genome-wide screening, that levels of FKBP5 were associated with response to two cytidine analogues, gemcitabine and cytosine arabinoside (AraC) (Li *et al*, 2008). Higher levels of expression of FKBP5 were associated with sensitivity and lower levels of expression were associated with resistance to gemcitabine and AraC. Furthermore, downregulation of FKBP5 desensitised pancreatic and breast cancer cell lines to several different classes of chemotherapeutic agents, including not only cytidine analogues but also taxanes, ironotecan, and etoposide (Li *et al*, 2008; Pei *et al*, 2009). In this case, the actions of FKBP5 on NF-*κ*B activation or hormone induction could not explain the increased chemoresistance in cells with decreased FKBP5 expression, suggesting the existence of other mechanisms by which FKBP5 regulates cell survival.

We recently identified FKBP5 as a negative regulator of AKT (Pei *et al*, 2009). Full activation of AKT requires phosphorylation at Thr308 within the activation loop of the kinase domain and Ser473 at a hydrophobic motif just C-terminal to the kinase domain. Thr308 is phosphorylated by pyruvate dehydrogenase kinase isozyme 1, mitochondrial (Alessi *et al*, 1997), while Ser473 is phosphorylated by mTOR complex 2 (Sarbassov *et al*, 2005). AKT activity is also negatively regulated by lipid or protein phosphatases. Two protein phosphatases have been shown to dephosphorylate AKT and negatively regulate AKT acivity. Protein phosphatase 2 (PP2, also known as PP2A) holoenzymes dephosphorylate Ser308 (Padmanabhan *et al*, 2009), while PH domain and leucine-rich repeat protein phosphatase 1 and 2 (PHLPP1 and 2) dephosphorylate Ser473 (Brognard and Newton, 2008). Therefore, the balance between kinases and phosphatases determines AKT activity. We found that FKBP5 functions as a scaffolding protein that enhances the PHLPP–AKT interaction and facilitates PHLPP-mediated dephosphorylation of AKT-Ser473. Downregulation of FKBP5 results in decreased PHLPP–AKT interaction and increased AKT phosphorylation. Therefore, FKBP5 might also function as a tumour suppressor in the AKT signalling pathway, similar to phosphatase and tensin homologue (PTEN). Indeed, we found that FKBP5 levels were low or absent in pancreatic cancer cell lines and tissue samples from patients with pancreatic cancer, correlating with increased AKT Ser473 phosphorylation. We also observed downregulation of FKBP5 in breast cancer cell lines. High levels of FKBP5 led to decreased AKT phosphorylation and increased chemosensitivity, whereas low levels of FKBP5 resulted in increased AKT phosphorylation and decreased chemosensitivity. These observations make FKBP5 a potentially important biomarker for sensitivity to chemotherapy, and variation in FKBP5 levels might determine patient responses to chemotherapy (Figure 2C and Table 1) (Pei *et al*, 2009). In addition, determination of levels of FKBP5 might provide insights that could help to select patients for different combination therapies with inhibitors targeting the AKT pathway (Pei *et al*, 2009).

## Conclusions

In summary, FKBP5 has altered expression levels in many different tumours. Through its influence on steroid receptor maturation, as well as NF-*κ*B and AKT signalling pathways, FKBP5 plays an important role in tumourigenesis and response to antineoplastic therapy (Figure 2 and Table 1). All of these observations raise the possibility that FKBP5 might be a biomarker for chemoradiosensitivity. On the basis of studies using lymphoblastoid cell lines from 300 individuals, we have observed large variations in FKBP5 expression that correlate with cellular sensitivity to gemcitabine and AraC (Li *et al*, 2008). Future studies will be needed to confirm the role of FKBP5 in predicting chemosensitivity in clinical samples. In addition, it would be important to investigate factors that are responsible for variation in FKBP5 expression in individuals. Common single-nucleotide polymorphisms (SNPs) in the *FKBP5* gene might contribute to variation in FKBP5 protein expression. Therefore, genotyping these SNPs and determining how they affect FKBP5 expression and, thus, drug sensitivity or tumourigenesis might be important next steps for this line of investigation. Additionally, the role of FKBP5 in tumourigenesis remains controversial. Whether FKBP5 functions as an oncogene or a tumour suppressor might depend on the tissue type and differences among pathways expressed in those tumours. For example, FKBP5 is found to be downregulated in pancreatic tumour tissue, while it is overexpressed in melanoma. These discrepancies need to be further clarified in the future using animal models.

Because of the role of FKBP5 in the regulation of multiple important biological pathways as discussed in this review, variation in FKBP5 could be of significance in many diseases beyond cancer, and in diseases such as diabetes (Tian, 2005). All of these possibilities will need to be investigated. Future studies will also be required to address questions such as how FKBP5 is regulated, and whether there are other pathways in which FKBP5 might be involved.

In summary, FKBP5 has multiple roles in the regulation of a variety of signalling pathways. Therefore, understanding the mechanisms underlying its regulation and its role in the development of cancer and in response to anti-neoplastic therapy could help make it possible to better individualise the treatment of human disease.

## Figures and Tables

**Figure 1:**
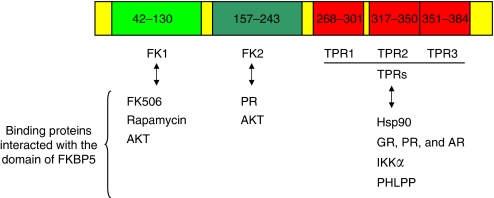
Structure of FKBP5 with its major domains that are critical for its function as well as with the list of its interactive proteins by domain. AKT=a serine/threonine protein kinase, also called PKB; AR=androgen receptor; FK=FKBP-type domain; GR=glucocorticoid receptor; Hsp90=heat shock protein 90; IKK*α*=I*κ*B kinase *α* subunit; PHLPP=PH domain and leucine-rich repeat protein phosphatases; PR=progesterone receptor; TPR=tetratricopeptide repeat.

**Table 1:** Table of relationship between FKBP5 levels in cancer and drug activity of antineoplastic agents

**Antineoplastic agent**	**Signalling pathway involved**	**Type of cancer**	**Change in FKBP51 expression**	**Alteration of drug susceptibility antineoplastic agent**	**Reference**
FK506, rapamycin	PR, GR	Breast cancer	Upregulated	Resistance	Le Bihan, Marsaud *et al* (1998)
FK506	AR	Prostate cancer	Upregulated	Resistance	Ni *et al* (2010)
Dexamethasone	GR	Myeloma	Upregulated	Resistance	Rees-Unwin *et al* (2007)
Rapamycin	NF-*κ*B	ALL, melanoma	Upregulated	Resistance	Romano, Avellino *et al* (2004); Avellino *et al* (2005)
Irradiation	NF-*κ*B	Melanoma	Upregulated	Resistance	Romano *et al* (2010)
Gemcitabine	AKT		Downregulated		
AraC	AKT		Downregulated		
Epitoside	AKT	Pancreatic cancer, breast cancer	Downregulated	Resistance	Li *et al* (2008); Pei *et al* (2009)
Texane	AKT		Downregulated		

Abbreviations: AKT=a serine/threonine protein kinase, also called protein kinase B; AR=androgen receptor; FKBP5= FK506 binding protein 5; GR=glucocorticoid receptor; NF-*κ*B=nuclear factor-*κ*B; PR=progesterone receptor.
